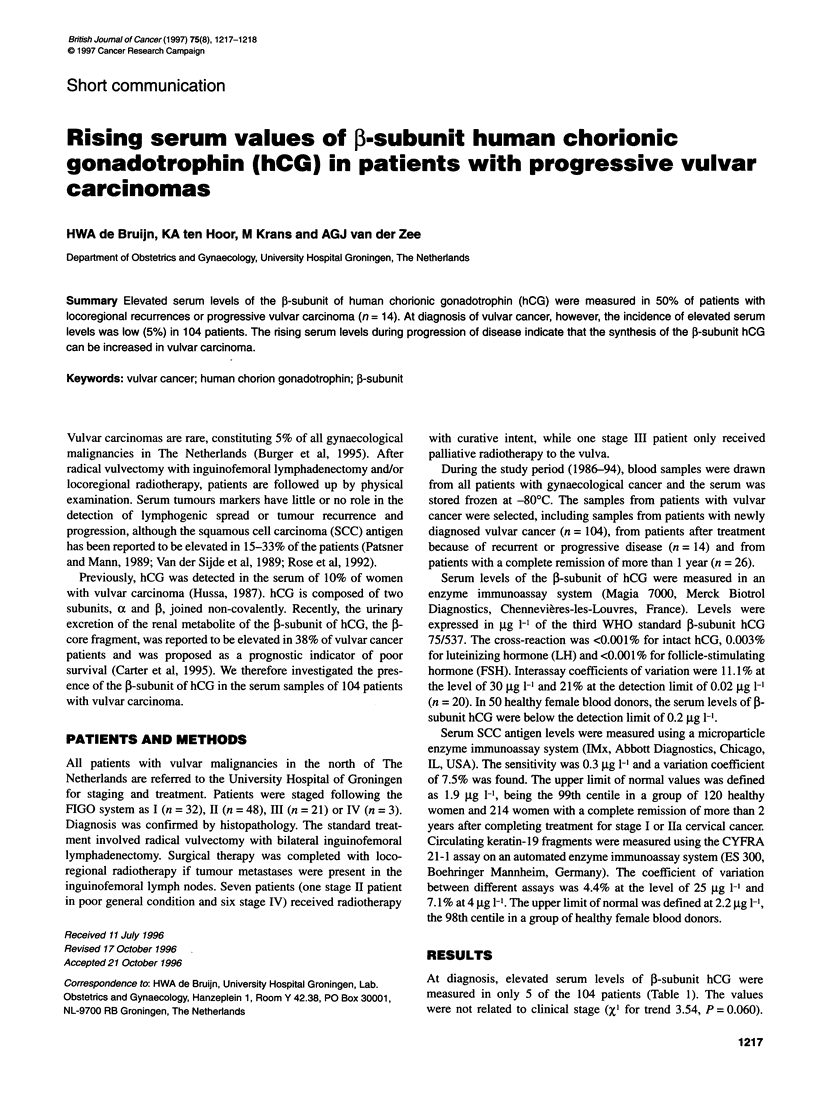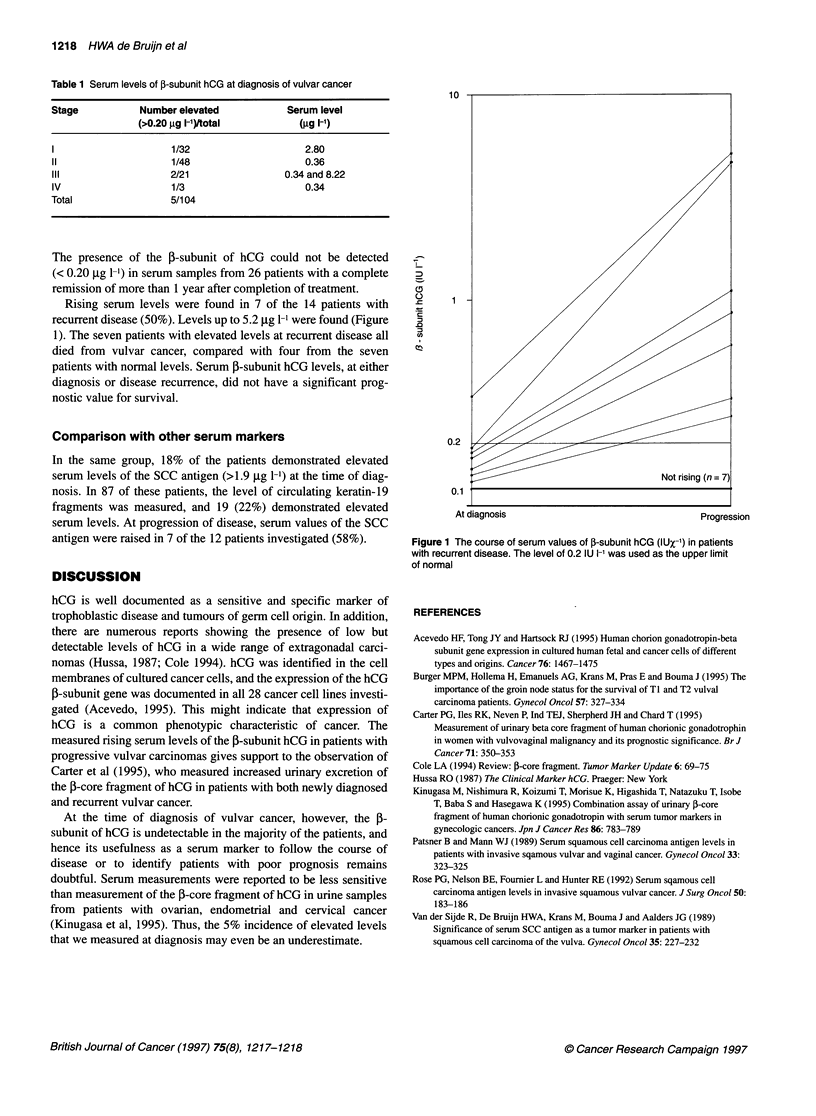# Rising serum values of beta-subunit human chorionic gonadotrophin (hCG) in patients with progressive vulvar carcinomas.

**DOI:** 10.1038/bjc.1997.208

**Published:** 1997

**Authors:** H. W. de Bruijn, K. A. ten Hoor, M. Krans, A. G. van der Zee

**Affiliations:** Department of Obstetrics and Gynaecology, University Hospital Groningen, The Netherlands.

## Abstract

Elevated serum levels of the beta-subunit of human chorionic gonadotrophin (hCG) were measured in 50% of patients with locoregional recurrences or progressive vulvar carcinoma (n = 14). At diagnosis of vulvar cancer, however, the incidence of elevated serum levels was low (5%) in 104 patients. The rising serum levels during progression of disease indicate that the synthesis of the beta-subunit hCG can be increased in vulvar carcinoma.


British Journal of Cancer (1997) 75(8), 1217–1218
© 1997 Cancer Research Campaign

Short communication

### Rising serum values of β-subunit human chorionic gonadotrophin (hCG) in patients with progressive vulvar carcinomas

**HWA de Bruijn, KA ten Hoor, M Krans and AGJ van der Zee**

Department of Obstetrics and Gynaecology, University Hospital Groningen, The Netherlands

**Summary** Elevated serum levels of the β-subunit of human chorionic gonadotrophin (hCG) were measured in 50% of patients with locoregional recurrences or progressive vulvar carcinoma ($n = 14$). At diagnosis of vulvar cancer, however, the incidence of elevated serum levels was low (5%) in 104 patients. The rising serum levels during progression of disease indicate that the synthesis of the β-subunit hCG can be increased in vulvar carcinoma.

**Keywords:** vulvar cancer; human chorion gonadotrophin; β-subunit

Vulvar carcinomas are rare, constituting 5% of all gynaecological malignancies in The Netherlands (Burger et al, 1995). After radical vulvectomy with inguinofemoral lymphadenectomy and/or locoregional radiotherapy, patients are followed up by physical examination. Serum tumours markers have little or no role in the detection of lymphogenic spread or tumour recurrence and progression, although the squamous cell carcinoma (SCC) antigen has been reported to be elevated in 15–33% of the patients (Patsner and Mann, 1989; Van der Sijde et al, 1989; Rose et al, 1992).

Previously, hCG was detected in the serum of 10% of women with vulvar carcinoma (Hussa, 1987). hCG is composed of two subunits, α and β, joined non-covalently. Recently, the urinary excretion of the renal metabolite of the β-subunit of hCG, the β-core fragment, was reported to be elevated in 38% of vulvar cancer patients and was proposed as a prognostic indicator of poor survival (Carter et al, 1995). We therefore investigated the presence of the β-subunit of hCG in the serum samples of 104 patients with vulvar carcinoma.

### PATIENTS AND METHODS

All patients with vulvar malignancies in the north of The Netherlands are referred to the University Hospital of Groningen for staging and treatment. Patients were staged following the FIGO system as I ($n = 32$), II ($n = 48$), III ($n = 21$) or IV ($n = 3$). Diagnosis was confirmed by histopathology. The standard treatment involved radical vulvectomy with bilateral inguinofemoral lymphadenectomy. Surgical therapy was completed with locoregional radiotherapy if tumour metastases were present in the inguinofemoral lymph nodes. Seven patients (one stage II patient in poor general condition and six stage IV) received radiotherapy with curative intent, while one stage III patient only received palliative radiotherapy to the vulva.

During the study period (1986–94), blood samples were drawn from all patients with gynaecological cancer and the serum was stored frozen at –80°C. The samples from patients with vulvar cancer were selected, including samples from patients with newly diagnosed vulvar cancer ($n = 104$), from patients after treatment because of recurrent or progressive disease ($n = 14$) and from patients with a complete remission of more than 1 year ($n = 26$).

Serum levels of the β-subunit of hCG were measured in an enzyme immunoassay system (Magia 7000, Merck Biotrol Diagnostics, Chennevières-les-Louvres, France). Levels were expressed in μg $l^{-1}$ of the third WHO standard β-subunit hCG 75/537. The cross-reaction was <0.001% for intact hCG, 0.003% for luteinizing hormone (LH) and <0.001% for follicle-stimulating hormone (FSH). Interassay coefficients of variation were 11.1% at the level of 30 μg $l^{-1}$ and 21% at the detection limit of 0.02 μg $l^{-1}$ ($n = 20$). In 50 healthy female blood donors, the serum levels of β-subunit hCG were below the detection limit of 0.2 μg $l^{-1}$.

Serum SCC antigen levels were measured using a microparticle enzyme immunoassay system (IMx, Abbott Diagnostics, Chicago, IL, USA). The sensitivity was 0.3 μg $l^{-1}$ and a variation coefficient of 7.5% was found. The upper limit of normal values was defined as 1.9 μg $l^{-1}$, being the 99th centile in a group of 120 healthy women and 214 women with a complete remission of more than 2 years after completing treatment for stage I or IIa cervical cancer. Circulating keratin-19 fragments were measured using the CYFRA 21-1 assay on an automated enzyme immunoassay system (ES 300, Boehringer Mannheim, Germany). The coefficient of variation between different assays was 4.4% at the level of 25 μg $l^{-1}$ and 7.1% at 4 μg $l^{-1}$. The upper limit of normal was defined at 2.2 μg $l^{-1}$, the 98th centile in a group of healthy female blood donors.

### RESULTS

At diagnosis, elevated serum levels of β-subunit hCG were measured in only 5 of the 104 patients (Table 1). The values were not related to clinical stage ($\chi^1$ for trend 3.54, $P = 0.060$).

*Received 11 July 1996*
*Revised 17 October 1996*
*Accepted 21 October 1996*

*Correspondence to*: HWA de Bruijn, University Hospital Groningen, Lab. Obstetrics and Gynaecology, Hanzeplein 1, Room Y 42.38, PO Box 30001, NL-9700 RB Groningen, The Netherlands

**Table 1** Serum levels of β-subunit hCG at diagnosis of vulvar cancer

| Stage | Number elevated (>0.20 μg $l^{-1}$)/total | Serum level (μg $l^{-1}$) |
|---|---|---|
| I | 1/32 | 2.80 |
| II | 1/48 | 0.36 |
| III | 2/21 | 0.34 and 8.22 |
| IV | 1/3 | 0.34 |
| Total | 5/104 | |

The presence of the β-subunit of hCG could not be detected (< 0.20 μg $l^{-1}$) in serum samples from 26 patients with a complete remission of more than 1 year after completion of treatment.

Rising serum levels were found in 7 of the 14 patients with recurrent disease (50%). Levels up to 5.2 μg $l^{-1}$ were found (Figure 1). The seven patients with elevated levels at recurrent disease all died from vulvar cancer, compared with four from the seven patients with normal levels. Serum β-subunit hCG levels, at either diagnosis or disease recurrence, did not have a significant prognostic value for survival.

### Comparison with other serum markers

In the same group, 18% of the patients demonstrated elevated serum levels of the SCC antigen (>1.9 μg $l^{-1}$) at the time of diagnosis. In 87 of these patients, the level of circulating keratin-19 fragments was measured, and 19 (22%) demonstrated elevated serum levels. At progression of disease, serum values of the SCC antigen were raised in 7 of the 12 patients investigated (58%).

### DISCUSSION

hCG is well documented as a sensitive and specific marker of trophoblastic disease and tumours of germ cell origin. In addition, there are numerous reports showing the presence of low but detectable levels of hCG in a wide range of extragonadal carcinomas (Hussa, 1987; Cole 1994). hCG was identified in the cell membranes of cultured cancer cells, and the expression of the hCG β-subunit gene was documented in all 28 cancer cell lines investigated (Acevedo, 1995). This might indicate that expression of hCG is a common phenotypic characteristic of cancer. The measured rising serum levels of the β-subunit hCG in patients with progressive vulvar carcinomas gives support to the observation of Carter et al (1995), who measured increased urinary excretion of the β-core fragment of hCG in patients with both newly diagnosed and recurrent vulvar cancer.

At the time of diagnosis of vulvar cancer, however, the β-subunit of hCG is undetectable in the majority of the patients, and hence its usefulness as a serum marker to follow the course of disease or to identify patients with poor prognosis remains doubtful. Serum measurements were reported to be less sensitive than measurement of the β-core fragment of hCG in urine samples from patients with ovarian, endometrial and cervical cancer (Kinugasa et al, 1995). Thus, the 5% incidence of elevated levels that we measured at diagnosis may even be an underestimate.

**Figure 1** The course of serum values of β-subunit hCG (IUχ⁻¹) in patients with recurrent disease. The level of 0.2 IU $l^{-1}$ was used as the upper limit of normal

### REFERENCES

Acevedo HF, Tong JY and Hartsock RJ (1995) Human chorion gonadotropin-beta subunit gene expression in cultured human fetal and cancer cells of different types and origins. *Cancer* **76**: 1467–1475

Burger MPM, Hollema H, Emanuels AG, Krans M, Pras E and Bouma J (1995) The importance of the groin node status for the survival of T1 and T2 vulval carcinoma patients. *Gynecol Oncol* **57**: 327–334

Carter PG, Iles RK, Neven P, Ind TEJ, Sherpherd JH and Chard T (1995) Measurement of urinary beta core fragment of human chorionic gonadotrophin in women with vulvovaginal malignancy and its prognostic significance. *Br J Cancer* **71**: 350–353

Cole LA (1994) Review: β-core fragment. *Tumor Marker Update* **6**: 69–75

Hussa RO (1987) *The Clinical Marker hCG*. Praeger: New York

Kinugasa M, Nishimura R, Koizumi T, Morisue K, Higashida T, Natazuku T, Isobe T, Baba S and Hasegawa K (1995) Combination assay of urinary β-core fragment of human chorionic gonadotropin with serum tumor markers in gynecologic cancers. *Jpn J Cancer Res* **86**: 783–789

Patsner B and Mann WJ (1989) Serum squamous cell carcinoma antigen levels in patients with invasive sqamous vulvar and vaginal cancer. *Gynecol Oncol* **33**: 323–325

Rose PG, Nelson BE, Fournier L and Hunter RE (1992) Serum sqamous cell carcinoma antigen levels in invasive squamous vulvar cancer. *J Surg Oncol* **50**: 183–186

Van der Sijde R, De Bruijn HWA, Krans M, Bouma J and Aalders JG (1989) Significance of serum SCC antigen as a tumor marker in patients with squamous cell carcinoma of the vulva. *Gynecol Oncol* **35**: 227–232

© Cancer Research Campaign 1997